# Non-cognitive does not work; we need a new name

**Published:** 2015-12-11

**Authors:** Marcel D’Eon

**Affiliations:** University of Saskatchewan, Saskatoon, Saskatchewan

Are you one of a growing number of medical educators uncomfortable using the term “non-cognitive” to describe a cluster of behavioural characteristics, personal attributes and interpersonal skills used for admissions? We are. Do you believe that ethical reasoning, situational judgments, complex communication skills, critical thinking, and even empathy and respect for all people are learned skills that require deep thinking and cognitive responses? We do, and so do many others.^1^,^2^ We need a new name!

With sincere apologies to William Shakespeare (and his fans) here is our parody of Juliet’s lament about names. (Romeo and Juliet Act II, Scene 2).^3^

*‘Tis thy name that is our enigma;*
*Thou art thy name, …..*

*…. O, take some other name!*
*But what name? that construct we call a rose*
*By any other name would never smell so sweet;*
*So would Non-cognitive, were he not so call’d,*
*Retain those vulgar values which he owns*
*Without that title? Non-cognitive, doff thy name,*
*For from that name which should be no part of thee*
*We turn all ourselves.*

We are advocating for a name change because non-cognitive does not work. However, there is some support for the *status quo* including a long history of the use of the term non-cognitive in many disciplines and lack of a popular alternative. In spite of common practice, though, few can argue that the name is appropriate or meaningful. All those elements we are trying to capture in an interview or reference letters or autobiographical essay all require thinking and are learned at one level or another. Farther back and in the K-12 world, researchers and policy makers were trying to identify those life skills and attributes that predicted and supported school success and labour market integration, what we think of as personality or character traits.^4^ They called them non-cognitive to distinguish them from the more academic and school generated marks and tests of mostly school subject achievement. Since then within medical education we have added other skills and attributes that go beyond personality and character traits (communication skills, etc.) and so the name ‘non-cognitive’ applies even less. To call these traits non-cognitive, to place them in a category with reflexes and basic physiological processes, does not work. To call them the opposite of what another group of attributes seems to be (cognitive – but even this is somewhat problematic) clouds and distorts our understanding of both their natures. All the constructs we are trying to measure for admissions are cognitive to some extent. This false dichotomy (cognitive vs. non-cognitive) creates an artificial and obstructive opposition where more nuance and subtlety are required to better describe this reality and advance the field. In addition, the term non-cognitive (not the good stuff we prize so highly and can measure so well) seems to privilege the cognitive skills. Non-cognitive does not work; we need another name.

*But what name?* Until we have a better name, even though we recognize that non-cognitive is a poor choice, there is nowhere to go. To overcome the inertia, we have created a few alternatives. We do not believe that any one of them is “the” answer but they do demonstrate that there are options which can free us up to start exploring. Notice that these suggestions are collections of terms. One word to unite them all would be wonderful but is certainly elusive and perhaps even seductive.

…. O, take some other name!

- Behavioural Attributes and Characteristics (BAC)
- Behavioural Attributes and Social Skills (BASS)
- Attributes and Psychosocial Skills (APS)

Let the quest begin!
